# Digital Attention-Related Augmented-Reality Game: Significant Correlation between Student Game Performance and Validated Clinical Measures of Attention-Deficit/Hyperactivity Disorder (ADHD)

**DOI:** 10.3390/children6060072

**Published:** 2019-05-28

**Authors:** Neha U. Keshav, Kevin Vogt-Lowell, Arshya Vahabzadeh, Ned T. Sahin

**Affiliations:** 1 Brain Power, 1 Broadway, Cambridge, MA 02142, USA; neha@brain-power.com (N.U.K.); kevin.vogt-lowell@brain-power.com (K.V.-L.); avahabzadeh@mgh.harvard.edu (A.V.); 2 Department of Psychiatry, Massachusetts General Hospital, Boston, MA 02114, USA; 3 Department of Psychology, Harvard University, Cambridge, MA 02138, USA

**Keywords:** autism, ASD, Google Glass, augmented reality, autism, special education, assistive technology, artificial intelligence, ADHD, attention, digital phenotyping, social-emotional learning, hyperactivity, serious games

## Abstract

As many as half of school children with autism spectrum disorder (ASD) exhibit symptoms of attention-deficit/hyperactivity disorder (ADHD), resulting in marked negative academic, social, and behavioral outcomes. The focus of the US Food and Drug Administration (FDA) on real-world data from novel digital sources, and the emergence of Current Procedural Terminology (CPT) codes to reimburse for digital monitoring and neurobehavioral testing suggest an increasing acceptance of the role of technology in augmenting clinical care and research. Empowered Brain is an augmented reality and artificial intelligence-based social-emotional communication aid for students with ASD. In this study, student performance on Empowered Brain is correlated to validated clinical measures of ADHD. Seven high school students with a diagnosis of ASD were recruited from a public high school. All students were assessed for severity of ADHD-related symptoms via three clinical gold-standard assessments, namely the Aberrant Behavioral Checklist (ABC), Social Responsiveness Scale 2 (SRS-2), and Teacher Report Form (TRF). Students used Empowered Brain over a one-week period. We measured the correlation of student in-game performance (as measured by point- and star-based rewards) relative to the hyperactivity subscale of the ABC (ABC-H), and the ADHD-subscale of the TRF. All seven students completed the study and managed to successfully use Empowered Brain. Students received a culminative total of 32 sessions, an average of 4.6 sessions per student (range 2–8). Student in-game performance demonstrated highly significant correlation relative to ABC-H (points: *p* = 0.0013; stars: *p* = 0.0013), and significant correlation to TRF ADHD scores (points: *p* = 0.012; stars: *p* = 0.012). No adverse effects were noted among students who used Empowered Brain. New technologies may herald novel ways of identifying and characterizing symptoms of ADHD in student populations. This study provides evidence that Empowered Brain in-game performance correlates with ADHD symptom severity in students with ASD. Larger samples are required to validate these findings, with more diverse participants that can also widen the generalizability of these findings to a broader range of brain conditions that manifest with inattention, impulsivity, and hyperactivity. Through further research, we may find that such technologies can help us to identify and longitudinally monitor such symptoms, and potentially aid in severity stratification and digital phenotyping.

## 1. Introduction

Autism spectrum disorder (ASD) is a life-long, neurodevelopmental condition predominantly characterized by challenges in social communication [1]. People with ASD have been shown to have worse educational [2,3], occupational [2,3], health [4], and social outcomes [5] than their neurotypical peers. Transition between adolescence and adulthood is an especially difficult developmental period for these individuals [6]. 

Up to half of individuals with ASD demonstrate symptoms associated with attention-deficit/hyperactivity disorder (ADHD) [7,8], manifestations that include symptoms from three key domains: motor hyperactivity, inattention, and impulsivity [1]. The combination of ADHD and ASD have been shown to be associated with greater negative outcomes that those seen in ASD populations alone, including worse academic performance and school-based behavioral problems [9]. The presence of ADHD-related symptomatology, even without a formal ADHD diagnosis, is associated with inferior academic function [10]. Research has highlighted that individuals with both ASD and ADHD have a greater number and severity of co-occurring mental health conditions than individuals with ASD alone [11]. Individuals with ASD and ADHD are also less likely to have their ADHD recognized and treated [12]. Additionally, these individuals respond worse to stimulant medication, the first-line medication for ADHD [13,14]. Monitoring of ADHD symptoms in this dual-diagnosed group is particularly challenging. 

At present, there are a variety of ways that ADHD can be diagnosed. Most typically, ADHD is a clinical diagnosis that is given by a behavioral specialist following the gathering of a clinical/educational history, observations of the individual, and collateral history from family members, educators, and other professionals. For almost 50 years, ADHD rating scales have supplemented this process, with current use favoring scales such as the Swanson, Nolan and Pelham Questionnaire, Fourth Edition (SNAP IV), Conners 3rd edition, and the Vanderbilt ADHD diagnostic rating scale (VADRS) (see reviews [15,16]). The diagnostic process focuses on identifying impairment in functioning in one of the three core symptom domains seen in ADHD, namely inattention, hyperactivity, and impulsivity. Unfortunately, there is little standardization of ADHD diagnosis, as there are different assessment processes between specialists, conflicting recommendations from professional medical societies, and subjective evaluative components that undermine diagnostic accuracy [17,18]. These factors are exacerbated by the highly heterogeneous nature of ADHD and, relevant to this report, are further complicated by the presence of co-occurring ASD. 

While a range of effective pharmaceutical treatments has been developed for ADHD, there is a lack of robust, ecologically valid measures to quantitatively assess and monitor the response to medical treatment. The importance of ecological validity should not be underestimated when dealing with individuals who have conditions that are diagnosed based on behavioral criteria. Obtaining patient-generated data from outside of clinical settings using new digital devices has been emphasized by FDA interest in the use of real world data (RWD) and real world evidence (RWE), to understand the potential benefits and adverse effects related to a medical intervention [19]. Digital tools can become mainstream ways to monitor and assess patients, including the use of technology to perform neuropsychological testing, as demonstrated by new Current Procedural Terminology codes for 2019. Current Procedural Terminology codes are used to identify specific billable services to health insurers and Medicare.

Attempts to develop quantitative assessment tools for ADHD has led to the development of several continuous performance tests (CPTs). These tests aim to objectively measure ADHD symptoms, especially in research settings. Examples of CPTs include Test of Variables of Attention (TOVA) and Connors CPT [18]. Typically, as part of a CPT assessment, participants are asked to differentially respond to a series of rapidly presented visual and/or auditory cues. Participants are asked to respond positively to some cues, but to withhold respond to others. These tests are often administered under strictly-controlled settings, with limited external distractions. CPTs may be particularly useful in assessing attention and impulsivity, as they assess an individual’s selective (correctly responding) and sustained attention (performance over time). However, these tests do not typically monitor the motor hyperactivity component of ADHD [18]. While inattention/impulsivity may present in a range of behavioral conditions, hyperactivity appears to be more specific to ADHD [18]. CPTs are not designed as stand-alone diagnostic instruments but may be used as part of a more comprehensive evaluation. In addition, concerns exist regarding the clinical utility of CPTs, as well as their limited specificity and sensitivity [20,21]. The strict environmental conditions in which CPTs are administered may have both poor ecological validity and limited generalizability compared to home and school environments [18]. Even within these real-world settings, attentional and behavioral demands vary based on context. meaning specific impairments can become more observable during certain situations.

Therefore, there is a need to develop novel technologies to assess ADHD-symptomatology. These technologies should objectively assess symptoms across all ADHD-symptom clusters (attention/impulsivity/hyperactivity) and should also demonstrate high ecological validity/ability to gather real-world data.

In this paper we report on the use of Empowered Brain during typical public high school classroom settings. Empowered Brain is a novel technology that combines computerized smartglasses, like Google Glass, and a series of game-like software modules. It is designed to be used by students as a wearable socio-affective aid as part of a daily school or home intervention program. Empowered Brain uses artificial intelligence to analyze video, audio, affective, and behavioral data and provides users with in-game rewards, such as points and stars, based on their performance. In this study of students with ASD, we report on the correlation between student ADHD symptoms and their in-game performance measurements. Student ADHD symptoms were determined through the use of validated scales, namely the hyperactivity subscales of the aberrant behavioral checklist (ABC) and the ADHD component of the Teacher Report Form (TRF) [22,23,24]. Both of these scales include items that assess all of the main symptom domains of ADHD. No additional staff, time, or equipment resources were provided during the time period that the study was conducted. 

Emerging research has demonstrated that Google Glass can be used as an assistive communicative and therapeutic technology for people with ASD, although both encouraging results and controversy have arisen. The Kushki lab at the University of Toronto has demonstrated the feasibility and usability of their unique system using Google Glass as a communication aid in ASD [25]. The Wall Lab at Stanford University (Autism Glass) has also shown that Glass may be a feasible social communication tool; however, they have been the subject of media controversy given concerns about lack of transparency, scientific rigor, and ethical practice [26]. Given the largely nascent nature of this technological field, and the vulnerable population that is being studied, it is important to ensure that robust scientific validation and ethical standards are maintained.

## 2. Methodology

### 2.1. Technological Background

**Figure 1:**
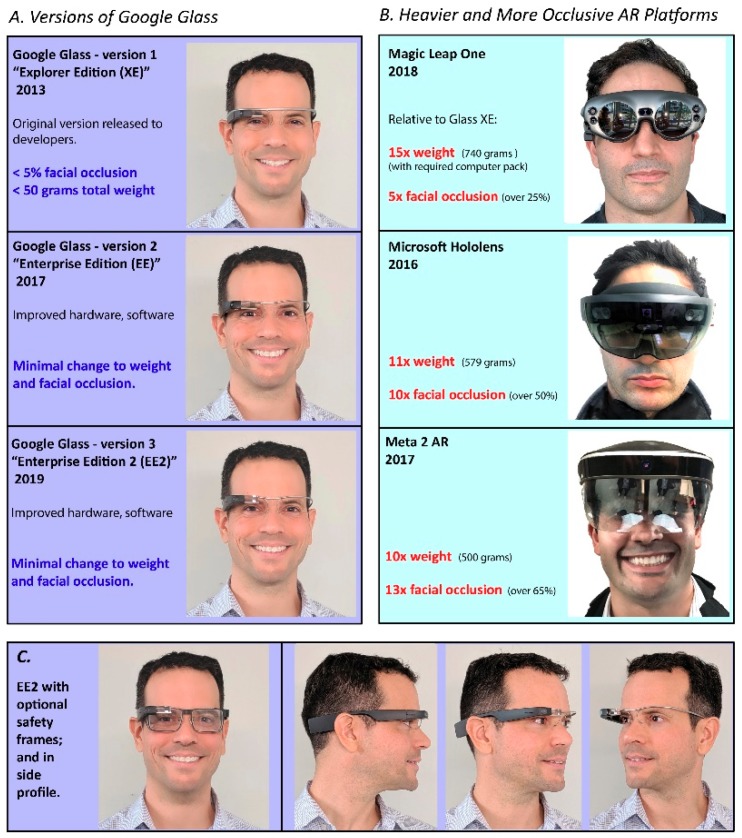
(**A**) Google Glass versions 1–3 (Explorer Edition, Enterprise Edition, Enterprise Edition 2) on an adult human face. Corresponding weight and facial occlusion characteristics are outlined. (**B**) Images of other augmented reality platforms (Magic Leap One, Microsoft Hololens, Meta AR) demonstrating increased weight and facial occlusion relative to Google Glass platforms. (**C**) Google Glass Enterprise Edition 2 with optional safety frames, and in side profile view without safety frames. Low occlusive characteristics are still maintained with addition of frames.

Empowered Brain is a wearable technology based on computerized glasses loaded with assistive software [27]. Empowered Brain provides users with real-time social communication and attention coaching [28,29,30,31]. The computerized glasses are most commonly Google Glass Explorer Edition (Figure 1).

Empowered Brain is worn on the face in a similar manner to glasses. Users of the device see visual guidance through a translucent display over their right eye and hear sounds through a right-sided bone conduction device. Empowered Brain consists of a variety of sensors, including a camera, microphone, accelerometer, gyroscope, and an inward-facing infrared sensor. Data from the sensors are gathered and are processed through AI-powered software. In-game rewards of points and stars are awarded based on performance and displayed in real-time to the user. These in-game rewards are awarded based on the same sensor data, and a number of points will result in a star reward that unlocks an “achievement” in the game and results in a temporary cartoon mask or embellishment of the partner’s face visible through the screen.

Empowered Brain has previously been found to be feasible to use across multiple studies [27,32]. The use of Empowered Brain has been found to be associated with improvements in social communication, irritability, and symptoms of ADHD [27,31,32]. The technology has also been found to be free of any major adverse effects [33] and capable of accurate real-time measurement of human behavior in brain-related conditions [34]. Empowered Brain has also been rated as desirable for school use [35], and has been studied in multiple longitudinal school-based intervention programs [28,29,32]. Empowered Brain was invented and engineered by Brain Power (Cambridge, MA, USA) and is based on novel software and hardware development partnerships afforded in part through relationships with X (formerly Google X, Mountain View, CA, USA), Affectiva (Boston, MA, USA), and Amazon (Seattle, WA, USA).

### 2.2. Study Design

A convenience sample of high school students were selected by educators to take part in the study. Criterion for inclusion was a current diagnosis of ASD according to school Individualized Education Program (IEP) records. An IEP is a collaboratively created document for students with special educational needs. An IEP outlines their educational needs and goals, while also setting out the interventions and resources that will be provided to the student. Educators were asked to assess students via an aberrant behavioral checklist (ABC) and the Social Responsiveness Scale (SRS-2). The ABC and SRS-2 are validated behavioral and social communication measures commonly used in ASD research, respectively [22]. Additionally, educators completed a TRF, a validated measure of behavioral and emotional problems in children [23,24]. All students who were referred, consented, and had educators complete their intake measures, were included in this study.

**Figure 2:**
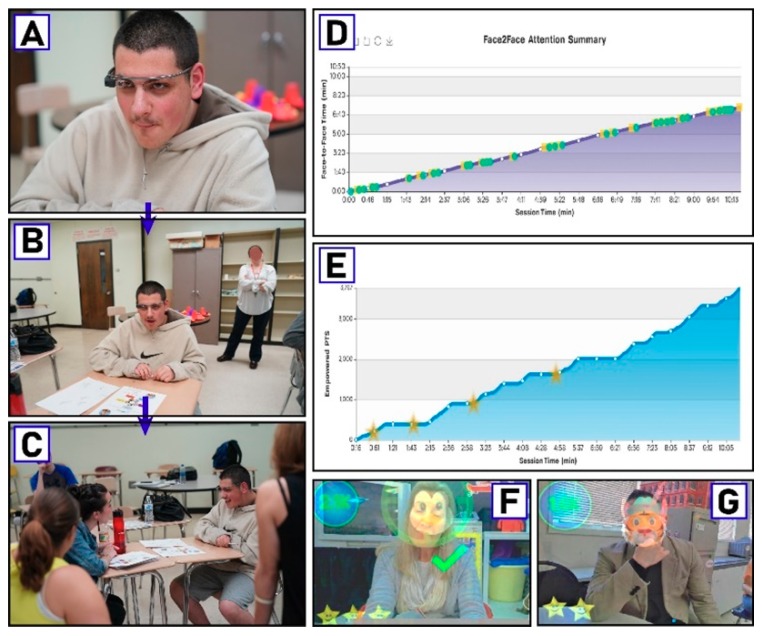
Overview of Empowered Brain as related to attention-deficit/hyperactivity disorder (ADHD) symptom stratification. (**A**–**C**) Non-study student wears Empowered Brain smartglasses and proceeds to engage with educator in live classroom setting. (**D**,**E**) Attention and game performance charts from Empowered Brain web account, a secure cloud-accessible data portal that reports student learning and performance. (**F**–**G**) Visual snapshots of what the student sees through the optical display during moments of achievement while using Empowered Brain. These snapshots are automatically recorded and available for teacher and student review through the student’s Empowered Brain account. Consent was obtained from all identifiable individuals to publish their photographs in publicly accessible research journals.

Over a one-week period of time, students were able to use Empowered Brain dependent on the availability of an educator to provide the intervention (Figure 2). The technology is designed to be studied and used in ecologically valid environments. Educators were therefore asked to incorporate the use of the technology in their classroom routine without the provision of additional support, such as staff, time, or other resources. Each session was approximately 10 min in duration and could occur up to a maximum of twice-daily during school days. Student performance was monitored by Empowered Brain device and software, and real-time feedback was given to students regarding points and stars obtained. The data collected from the session, including student performance, is stored and analyzed in a web-accessed portal. This allows teachers to generate summary reports of student performance in a format compatible to IEP reports.

The intervention involved a dyadic interaction between the student and the educator. The student wore Empowered Brain in a similar manner to glasses, and the educator sat in front of the student. At the start of the intervention, the educator powered on the system and loaded the Empowered Brain module. The educator then provided the system to the student and initiated an unstructured academic discussion with him/her regarding a variety of recommended topics, such as homework assignments, classroom projects, or behaviors in relation to home and/or peers. During this time, Empowered Brain monitored the student-educator interaction and provided the student with appropriate visual and auditory guidance to help direct his/her attention to emotional and social communication cues.

As the participant followed the provided digital guidance, for instance by increasing and sustaining attention to the central facial area of the educator, he/she received in-game rewards.

During the interaction, improvement in student social communication skills is met with increased game difficulty, whereas lapses in attention or reduced markers of social engagement are met with increased guidance/decreased game difficulty.

### 2.3. Outcome Measures

The main assessed measures used in this study were those from the hyperactivity subscale of the Aberrant Behavior Checklist (ABC–H) [36] and the combined ADHD subscale of the TRF [37]. The ABC consists of five subscales, namely: (1) Irritability, Agitation, Crying; (2) Lethargy/Social Withdrawal; (3) Stereotypic Behavior; (4) Hyperactivity/Noncompliance; and Inappropriate Speech. The ABC-H is a particularly useful measure of ADHD symptoms in ASD populations [38]. The ABC-H has been used extensively as a screening and outcome measure in ADHD-related studies in ASD populations [13,27,31,38,39,40,41,42]. The ABC-H contains items that assess ADHD-related symptoms, including hyperactivity, impulsivity, inattention, and defiance [36]. The TRF is an empirically-validated syndrome scale capable of identifying emotional and behavioral problems in school-age children [43]. It has six-DSM orientated scales that have been identified by experts as being consistent with DSM-5 diagnostic categories. The TRF refers to “problem” symptoms, namely depression, anxiety, somatic, thought, and attention-deficit/hyperactivity. The TRF has been validated across many societies and countries [44], and has been used in research studies into ADHD [45].

### 2.4. Participants and Setting

There were seven participants in this research study (M = 6: F = 1) (Table 1). Mean age of the participants was 15.6 years (range 14–18 years). Participants identified as being white (n = 5), African-American (n = 1), and Hispanic/Latino (n = 1). Participant language ability ranged from non-verbal to fully verbal. Participants received the intervention concurrently with their usual lessons, in their usual classroom setting, as facilitated by their usual educators. Educators received training on how to use Empowered Brain.

### 2.5. Consent and Institutional Review Board Status

Research of Empowered Brain running on multiple head-worn computing devices and its use with children and adults with ASD was approved by Asentral, Inc., IRB, an affiliate of the Commonwealth of Massachusetts Department of Public Health (protocol no. BPSCOM2, date of approval 17 November 2017). The study was performed in accordance with relevant guidelines and regulations, and in accordance with the Helsinki Declaration. Written informed consent was obtained from all parents/legal guardians of all minors involved in this study and consent was provided by all capable participants. Consent to conduct this research was also obtained from all educators involved in the study. Written informed consent was obtained for the publication of photographs and other identifiable information of participants.

## 3. Results

Participants (n = 7) had a mean SRS-2 T-score of 78.3 (range 73 to >90), and a mean ABC-H of 7.9 (range 0–20). Participants’ mean TRF ADHD raw score was 7.7 (range 0–15) with a mean TRF ADHD T-score of 56.7 (50–65). Participant ABC-H score and TRF ADHD raw scores were significantly correlated (*p* = 0.01).

Participant in-game performance was successfully gathered from all participants. Participants had a mean of 4.6 Empowered Brain sessions (total number 32, range 2–8). All participants were able to demonstrate an ability to obtain rewards.

**Figure 3:**
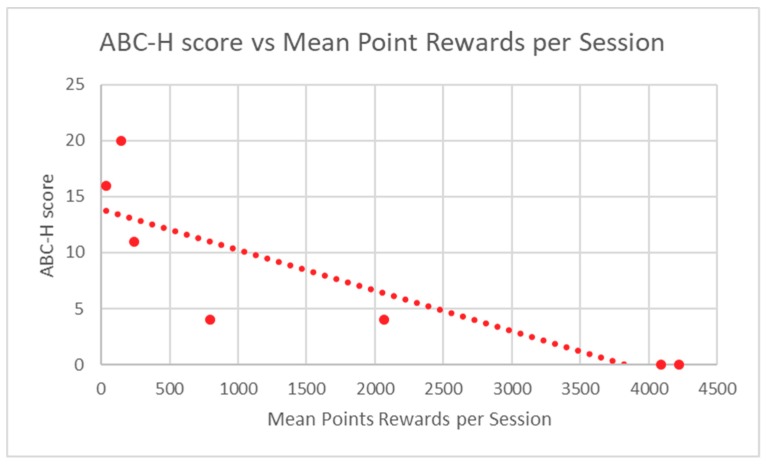
ABC-H Score vs. Mean Point Rewards Per Session.

**Figure 4:**
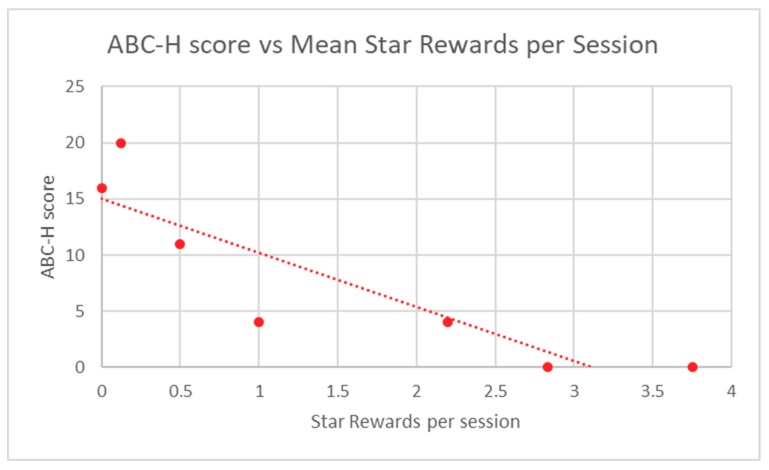
ABC-H score vs. Mean Star Rewards per Session.

**Figure 5:**
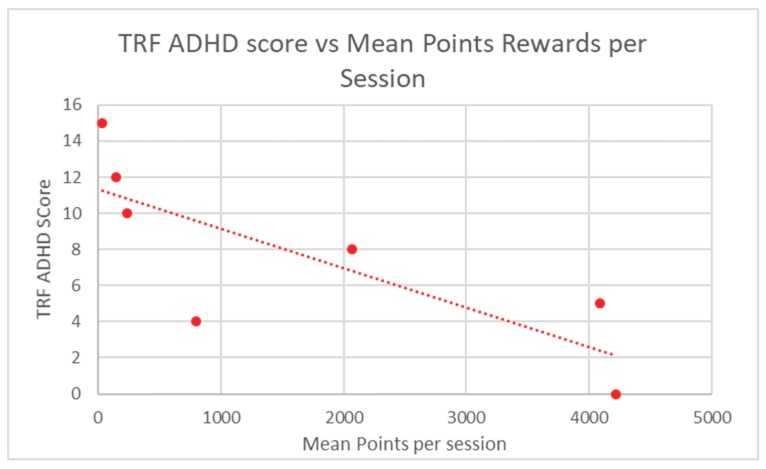
TRF ADHD score vs. Mean Star Rewards per Session.

**Figure 6:**
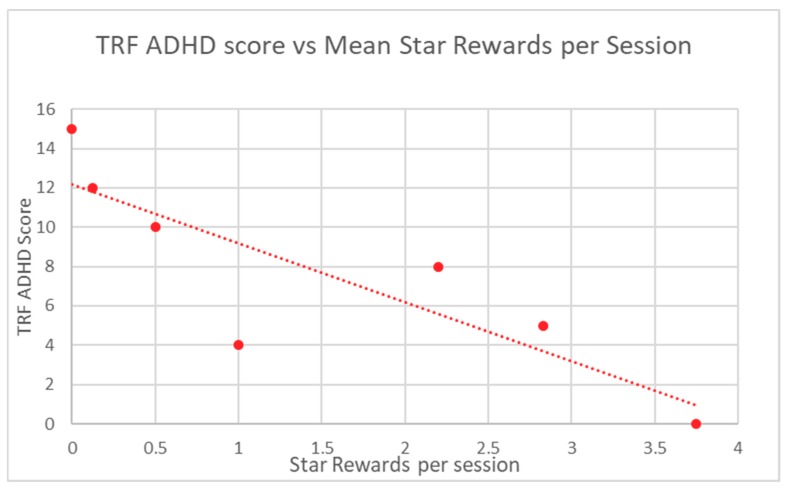
TRF ADHD score vs. Mean Star Rewards per Session.

Participant in-game performance, as assessed by stars and points rewards, was correlated to ABC-H and TRF ADHD raw scores through the calculation of a Spearman Rank correlation coefficient. A *p*-value was determined from this score. Participant in-game performance demonstrated highly significant correlation to baseline ABC-H for both game points (*p* = 0.0013) and game stars (*p* = 0.0013) (Table 2). TRF ADHD raw scores were also significantly correlated to student in-game performance in regard to points (*p* = 0.012) and stars (*p* = 0.012) (Table 3). Mean participant performance is displayed in Figure 3 and Figure 4 (ABC-H) and Figure 5 and Figure 6 (TRF ADHD). Given that the game points and star measures were not independently-related game components, and were directly linked to one another, statistical correction was not deemed necessary. No adverse effects were noted among students who used Empowered Brain.

## 4. Discussion

New technologies may herald novel ways of identifying and characterizing symptoms of ADHD in student populations. Over 6.4 million children and adolescents in the US have been diagnosed with ADHD [46], and the condition currently impacts over 10 million US adults [47]. Despite this, research suggests that ADHD symptoms continue to be under-recognized despite leading to adverse educational and health outcomes. Technology may allow for a highly scalable and objective assessment of ADHD symptoms. While technology could augment traditional subjective approaches, its utility and ecological validity must be assessed.

This study provides supportive evidence that Empowered Brain is a technology that can be used by students and teachers within a regular classroom setting. The usability of this system was demonstrated despite methodologically-imposed limitations designed to ensure high ecologically validity. These limitations include student use of the technology, as facilitated by their own regular teachers, during their regular lessons/school day, and within their own classrooms. No additional time, staff, or fiscal resources were provided except for initial teacher training. The study demonstrated that the in-game performance of the students was significantly correlated to two clinically validated measures of ADHD symptom severity. The findings were highly significant in the case of the ABC-H measure.

While this study has a sample size of only seven participants, to the authors knowledge, this is the first report of the use of assessment of ADHD symptom severity through the use of socio-emotional game performance on smartglasses. The data were collected over a one-week period and involved a total of 32 game sessions, each lasting 10 min in duration, providing for 320 min of use. Given these findings, it would be important to conduct further studies that address questions raised by this study. Firstly, further research should attempt to reproduce the results with a larger student sample size. Secondly, use of a broader age range of students across different school types could aid the generalizability of the findings across a broader range of students. Thirdly, a more detailed assessment of the presence of confounding disorders such as anxiety and depression should be undertaken, especially as they influence social interaction.

Future work will focus on gathering data on the use of Empowered Brain in non-ASD populations who may experience challenges in inattention, hyperactivity, and impulsivity. As symptoms of inattention and impulsivity occur across a broad transdiagnostic range of conditions, the possibility of using this technology across a much broader student and clinical population can also be considered.

Despite these limitations, Empowered Brain has already been shown to improve social communication and behavior in people with ASD when used as a daily socio-affective coaching tool. While providing this intervention through game-like experiences, Empowered Brain collects and analyzes important information about the user through data-gathering sensors and artificial intelligence-based analysis. While further research is warranted, the potential for Empowered Brain to be able assess ADHD symptom load would be an interesting possibility. A digital tool that is practical to use while providing quantitative stratification of ADHD symptom severity could help identify the needs of students while not unduly impacting school resources. This technological approach may have utility to further aid digital phenotyping efforts seen in mental health conditions [48,49].

Additional research is warranted to determine whether the system could function in a longitudinal monitoring role. Empowered Brain has already demonstrated its ability to generate real-world data and real-world evidence in a series of research studies. Gathering more robust evidence to validate the ability of Empowered Brain to objectively and accurately measure ADHD symptoms is important. This validation could allow for the use of such technology as an outcome measure tool in ADHD clinical research. The ability to do this in a quantitative and standardized manner is highly desirable. We should also consider that there is evidence Empowered Brain can improve ADHD symptoms. Therefore, there is a possibility that participant ADHD symptom load may have changed during their use of Empowered Brain in this study (mean number of sessions: 4.6). We should also note that prior attempts to use CPT as an objective measure of ADHD symptoms have often focused on the measurement of attention and impulsivity, with limited assessment of hyperactivity/motor behavior. This is important given inattention and impulsivity are commonly encountered transdiagnostic symptoms across many behavioral conditions, whereas hyperactivity may be much more specific to ADHD [18]. The Empowered Brain captures user performance and interaction across all three ADHD symptom domains, including hyperactivity, allowing the system to provide a more comprehensive approach to ADHD assessment as compared to most CPTs. 

The research and development of technologies that can quantify behavior represents a broader trend to use technology to obtain patient-generated data from non-clinical settings. This data can help to understand the risks and benefits associated with new health interventions [RWE). Additionally, digital patient monitoring and neuropsychological testing have been introduced as new codes for the Current Procedural Terminology code set for 2019, allowing them to be billable services for health insurers and Medicare.

## 5. Conclusion

This study demonstrates that Empowered Brain in-game performance correlates with, and may be predictive of, ADHD-related symptoms in students with ASD. These preliminary findings were obtained in real world educational settings, without the provision of any additional school resources. Further research with larger participant numbers is required to replicate these findings. There is potential for this type of technology not only to aid identification and longitudinal monitoring of such symptoms, but also to provide for an assessment of severity level and subtype. Larger and more diverse samples will be required to improve the generalizability of the present findings to a broader range of brain conditions that manifest with inattention, impulsivity, and hyperactivity.

## Figures and Tables

**Table 1:** Participant Demographics.

Participant	Age	Gender	Ethnicity	Grade	Educatior-Rated Verbal Ability
1	15	M	White	9th	Full
2	15	F	Black/African American	9th	Minimal
3	14	M	Hispanic/Latino	9th	Non-verbal
4	15	M	White	9th	Full
5	16	M	White	10th	Full
6	18	M	White	10th	Full
7	16	M	White	10th	Full

**Table 2:** Mean Participant ABC-H Score vs In-game Performance (Points & Stars Rewards).

Participant	ABC-H Score	Mean Points	Mean Stars
1	20	143.4	0.125
2	0	4089.3	2.83
3	11	240	0.5
4	4	2067	2.2
5	4	797.6	1
6	16	33.5	0
7	0	4222	3.75
	Spearman Rho	−0.95	−0.95
	*P*-Value	0.0013 **	0.0013 **

** highly significant < 0.01).

**Table 3:** Mean Participant Teacher Report Form (TRF) attention-deficit/hyperactivity disorder (ADHD) Raw Score vs In-game Performance (Points & Stars Rewards).

Participant	TRF ADHD Raw Score	Mean Points	Mean Stars
1	12	143.4	0.125
2	5	4089.3	2.83
3	10	240	0.5
4	8	2067	2.2
5	4	797.6	1
6	15	33.5	0
7	0	4222	3.75
	Spearman Rho	−0.89	−0.89
	*P*-Value	0.012 *	0.012 *

* significant < 0.05.
